# Rapid Identification of Known and New RNA Viruses from Animal Tissues

**DOI:** 10.1371/journal.ppat.1000163

**Published:** 2008-09-26

**Authors:** Joseph G. Victoria, Amit Kapoor, Kent Dupuis, David P. Schnurr, Eric L. Delwart

**Affiliations:** 1 Department of Molecular Virology, Blood Systems Research Institute, San Francisco, California, United States of America; 2 Department of Laboratory Medicine, University of California, San Francisco, California, United States of America; 3 Viral and Rickettsial Disease Laboratory, Division of Communicable Disease Control, California State Department of Public Health, Richmond, California, United States of America; Columbia University, United States of America

## Abstract

Viral surveillance programs or diagnostic labs occasionally obtain infectious samples that fail to be typed by available cell culture, serological, or nucleic acid tests. Five such samples, originating from insect pools, skunk brain, human feces and sewer effluent, collected between 1955 and 1980, resulted in pathology when inoculated into suckling mice. In this study, sequence-independent amplification of partially purified viral nucleic acids and small scale shotgun sequencing was used on mouse brain and muscle tissues. A single viral agent was identified in each sample. For each virus, between 16% to 57% of the viral genome was acquired by sequencing only 42–108 plasmid inserts. Viruses derived from human feces or sewer effluent belonged to the *Picornaviridae* family and showed between 80% to 91% amino acid identities to known picornaviruses. The complete polyprotein sequence of one virus showed strong similarity to a simian picornavirus sequence in the provisional *Sapelovirus* genus. Insects and skunk derived viral sequences exhibited amino acid identities ranging from 25% to 98% to the segmented genomes of viruses within the *Reoviridae* family. Two isolates were highly divergent: one is potentially a new species within the orthoreovirus genus, and the other is a new species within the orbivirus genus. We demonstrate that a simple, inexpensive, and rapid metagenomics approach is effective for identifying known and highly divergent new viruses in homogenized tissues of acutely infected mice.

## Author Summary

Viral surveillance programs aim to identify circulating viruses to safeguard the public and livestock from viral outbreaks. Occasionally, samples suspected of harboring a virus cause severe disease in laboratory animals, but the identity of the virus eludes researchers. Here, we applied a simple viral discovery technique to identify viruses directly from the tissues of inoculated symptomatic mice and found a single virus in each sample using a rapid viral particle purification and random nucleic acid amplification method. Two viruses appear to be closely related to the members of the *Picornaviridae* family. In three other samples, originally collected from pools of crushed mosquitoes and the brain of a sick skunk, a known and novel viruses related to members of the *Reoviridiae* family were identified. Reoviruses are considered major pathogens of livestock. Our studies provide the groundwork for further analysis of the prevalence and pathogenesis of these divergent viruses and illustrate the ease with which new viral species can be identified in tissues of acutely infected animals.

## Introduction

Clinical laboratories are rapidly adopting viral species-specific nucleic acid amplification for virus identification, thereby increasing the sensitivity of detection and reducing the time needed for diagnosis. Although widely successful, these methods are limited for detecting divergent viruses due to their high specificity. Failure rates in determining the etiological cause of disease are varied. For example, the rate for encephalitis is between 30–85% [reviewed in [1]], approximately 12% for acute flaccid paralysis [2], and for non A-E hepatits between 18–62% [reviewed in [3]]. In cases where routine screening fails, newer technologies are now being employed. Prominent among these are microarrays and sequence-independent amplification and sequencing of viral nucleic acids [4],[5],[6],[7],[8],[9],[10],[11],[12],[13],[14].

Viral microarrays can be used to screen for all viral families simultaneously and have been used successfully to detect novel human rhinoviruses [10], human coronaviruses [10], and a human gamma retrovirus closely related to mouse retroviruses [14]. Microarrays require sufficient sequence similarities between virus and array oligonucleotides for hybridization to occur, making the detection of highly divergent viruses problematic. Sequence-independent amplification of nuclease protected viral particles [15] abrogates the need for *a priori* sequence information, allows the detection of viruses recognizable through their protein sequence homologies to known viruses and has successfully been used to identify novel human and bovine parvoviruses [4],[8],[15], polyomaviruses [7],[16],[17], anelloviruses [5], an arenavirus [18], a dicistrovirus associated with honey bee colony collapse disorder [19], and a seal picornavirus [9].

In this study we utilized sequence-independent amplification of partially purified viral nucleic acid from mouse tissue followed by low-scale shotgun sequencing to quickly identify the viral agents in five samples negative by tests available at the time of inoculations. Of the five viruses identified, two belonged to the *Picornaviridae* family, and three to the *Reoviridae* family.

## Materials and Methods

### Sample preparation and viral culture

VRDL1 isolate was originally derived from sewer effluent from Hamilton, Montana in 1959 and passaged five times through intracerebral inoculation of suckling mice. Subsequently, the viral stock was adapted to growth in primary rhesus monkey kidney cells, and mice were re-inoculated by intramuscular injection. Moribund mice were sacrificed; muscle tissue was collected. VRDL2 was isolated from the muscle of a moribund mouse inoculated with a fecal suspension of a 17 year old female suffering flu like symptoms in 1960. VRDL3 and VRDL4 were isolated from the brains of suckling mice paralyzed after inoculation of ground tissue from a pool of *C. tarsalis* mosquitoes or a pool of mosquitoes from multiple species, respectively. VRDL5 was isolated from the brain of a suckling mouse paralyzed after inoculation of filtered, ground skunk brain tissue in 1974. For all samples, 0.5–1.0 gm of either muscle or brain was placed in a sterile mortar, sprinkled with sterile alumdum and ground to a pulp. The pulp was then suspended in 0.75% bovine albumin in buffered saline (BABS) at pH 7.2, to either 10% or 20% weight-to-volume. This suspension was then centrifuged @2500 RPM at 4°C for 20 minutes. The supernatant was removed and 20,000 U/mL Penicillin-Streptomycin was added to 1% by volume. Suspensions were then aliquoted and frozen at −70°C.

### Isolation of viral nucleic acid

Cellular debris and bacteria were removed from mouse tissue homogenates by filtration through 0.45 µM filter (Millipore). Filtered viral particles were then pelleted by centrifugation at 22,000× g for 2 hrs at 8°C and resuspended in Hanks buffered saline solution (Gibco, BRL). Non-particle protected (naked) DNA and RNA was removed by digestion with a cocktail of DNase enzymes consisting of 14 U of turbo DNase (Ambion), 20U benzonase (Novagen) and 20U of RNase One (Promega) at 37°C for 90 minutes in 1× DNase buffer (Ambion). Remaining total nucleic acid was then isolated using Qiamp Viral RNA isolation kit (Qiagen) according to manufacturer's protocol. Purified viral RNA was protected from degradation by addition of 40U of RNase inhibitor (Invitrogen) and stored at −20°C.

### Reverse transcriptase priming and amplification of nucleic acids

Viral cDNA synthesis was performed by incubation of the extracted viral RNA/DNA with 100pmol of primer K-8N (GAC CAT CTA GCG ACC TCC ACN NNN NNN N) [13], and 0.5mM of each deoxynucloside triphosphate (dNTP) at 75°C for 5 min. Subsequently, 5U of RNase inhibitor, 10mM dithiothreitol, 1× first-strand extension buffer, and 200U of SuperScript II (Invitrogen) were added to the mixture and incubated at 25°C for 5 min, followed by 37°C incubation for 1 hr. In order to generate products with the fixed portion of primer K-8N on the opposite strand of the cDNA, single round priming and extension was performed using Klenow fragment (New England Biolabs). 20 µl of cDNA was heated to 95°C for 2 min and then cooled to 4°C in the presence of 20pmol of primer K-8N in 1× klenow reaction buffer. 5U of Klenow fragment were added and incubated at 37°C for 60 min.

PCR of extension products was performed using 5 µl of the reaction described above in a total reaction volume of 50 µl containing 2.5mM MgCl_2_, 0.2mM dNTPs, 1× PCR Gold buffer, 0.8 µM primer K (GAC CAT CTA GCG ACC TCC AC), and 0.94U of AmpliTaq Gold. Temperature cycling was performed as follows: 1 cycle of 95°C for 5 min, 5 cycles of denaturing at 95°C for 1 min, annealing at 59°C for 1 min, extension at 72°C for 1 min, 33 cycles of denaturing at 95°C for 20 sec, 59°C for 20 sec, 72°C for 1 min + 2 sec each cycle. An additional extension for 7 min at 72°C was added to the end of the run. Products were distinguished as a smear by agarose gel electorphoresis. Fragments larger than 500bp were isolated (Qiagen) and subcloned into pGem T-easy vector (Invitrogen) for sequencing.

### Comparative sequence analysis

Reference sequences were obtained from NCBI and edited using GENEDOC software. Accession numbers for sequences used analyses are as follows: Picornaviruses; Simian picornavirus 1 (AY064708), Simian picornaviruses VP1 (AAL69622-AAL69637), Human enteroviruses 89 (AY697459), 91 (AY697461), 83 (AY843301), 94 (EF107097), and 70 (DQ201177), 94 (EF107097), 70 (DQ201177), Coxsackie A viruses 5 (AF081296), 10 (AF081300), 3 (AF41342), 11 (AF499636), 15 (AF465512), 19 (AF08138) and 1 (AF499635), Coxsackie B4 virus (AF311939), Human Echoviruses 30 (AF311938) and 2 (AY302545), Bovine Enteroviruses (AF123433, D00214), Porcine Enteroviruses (AF363453, AF406813), Duck Picornavirus TW90A (YP164335). Reoviruses; Eyach virus (NC_003696-NC_003707), Colorado Tick and Fever Virus (NC_004180_004191). Accession numbers for sequences described in this study are as follows: SV49 strain VRDL1 (EU789367), skunk orthoreovirus (EU789368-EU789373), Eyach virus strain VRDL4 (EU789374-EU789390), and California mosquito pool virus (EU789391-EU789395). Sequence alignments were generated using the CLUSTAL_W package with the default settings. CLUSTAL_W alignments were used to generate phylogenetic trees in MEGA 4 using either neighbor-joining, maximum likelihood or maximum parsimony with bootstrap values calculated from 1000 replicates. The generated phylogenetic trees were visualized using the program MEGA 4.

## Results

### Summary of Viruses

As part of infectious agent surveillance programs, sewer effluent, ground mosquito pools and skunk brain tissue collected between 1959 and 1980 by the California Department of Public Health were used to inoculate suckling mice intramuscularly or intracerebrally. In five samples, infectious agents could not be indentified in brain or muscle homogenates using available techniques, although the inoculated mice exhibited severe pathology. In this study, we used sequence-independent amplification of viral particle protected nucleic acids following nuclease digestion [8],[13],[15] of infected mouse tissue to identify a viral agent in each of these samples. These viruses are summarized in Table 1. In total, 47% of all plasmid subclones were viral in origin, indicative of a high viral concentration after filtration and nuclease treatment for both brain and muscle tissues. Genomic coverage ranged from 55–57% for picornaviruses (∼7–9 kb viral genomes), but only 16–46% for the larger genome reoviruses (∼19–29 kb) (**Table 1**). Viral sequences were further characterized through phylogenetic analysis.

**Table 1:** Summary of Viral Sequences

Lab ID (virus name)	Source^1^	Year	Nearest Blastx (Amino Acid Ident.)	Genomic Region Amplified^2^	% Genome Seq.	Viral Clones
VRDL1 (SV49 -VRDL1)	20% MM	1955	Simian Picornavirus: NC_004451 (80%)	5′ UTR, **VP4, VP2, VP3, VP1**, 2A, 2C, 3A, **3C**, 3D	55	43/46^3^
VRDL2 (CoxA22)	20% MM	1978	Coxsackievirus A22: AF499643 (89%)	5′ UTR, **VP4, VP2**, VP3 VP1, 3A, **3B, 3C**, 3D	57	33/42
VRDL3 (CMPV)	20% MB	1974	Orbivirus (25%)	Seg. 2, 3, 4, 6, 7, 9	17	33/108
VRDL4 (Eyach)	10% MB	1980	Eyach Virus: NC_003696-707 (98%)	Seg 1, 2, 3, 4, 5, 7, 8, 9	47	34/61
VRDL5 (Skunk Orthoreovirus)	20% MB	1974	Mammalian Orthoreovirus (50%)	Seg L2, M2, M3, S3, S4	16	10/66

1 MM = Suspension from injected mouse muscle, MB = Intracerebral injection of neonatal mouse brain suspension.

2 Bold indicates at least 95% of genomic region sequenced.

3 The complete VRDL1 polyprotein and 3′-UTR was obtained by linking fragments via PCR and 5′- and 3′-RACE.

### Picornaviruses

#### Enterovirus

**Figure 1:**
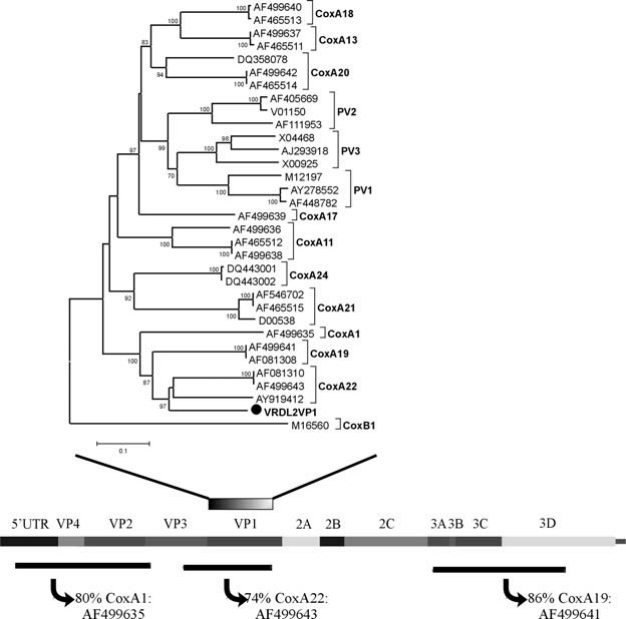
Divergent CoxA22 genotype. Percent nucleotide identity of three VRDL2 fragments (black lines) to closest Blastn enterovirus genome. VP1 amino acid (shaded box) sequence used to create neighbor-joining phlyogenetic tree of HEV-C with bootstrap values from 1000 replicates.

According to California Department of Public Health records, sample VRDL2 produced flaccid paralysis in injected suckling mice with diffuse myositis of trunk and limb muscles. These symptoms are consistent with Coxsackie A virus infection. However, all serological tests were negative for enteroviruses [20]. In the current study, purified viral nucleic acids randomly amplified from VRDL2-infected mouse muscle confirmed infection with a moderately divergent Coxsackie A virus (CoxA). Pair-wise sequence comparisons of VRDL2 revealed between 74–86% nucleotide identity and 72–98% amino acid identity to CoxA1, CoxA19 and CoxA22, which comprise a monophyletic cluster within the human enterovirus C species (HEV-C). Coxsackie virus serotypes have been shown to correlate with their phylogenetic groupings based on sequence similarity, with approximately >75% nucleotide identity and >88% amino acid identity in the VP1 corresponding to the same serotype [21]. To determine the phylogenetic relationships between VRDL2 and serotypes of HEV-C several phylogenetic reconstruction algorithms (maximum likelihood, maximum parsimony and neighbor-joining) were used to compare HEV-C VP1 sequences (**Fig. 1**). At least three representatives, when available, from each member of HEV-C were used; a single member of human enterovirus B was included as an outgroup. As suggested by amino acid identity, VRDL2 segregated with CoxA22 with bootstrap values between 71–98%, as a basal member of the CoxA22 serotype.

#### Sapelovirus

**Figure 2:**
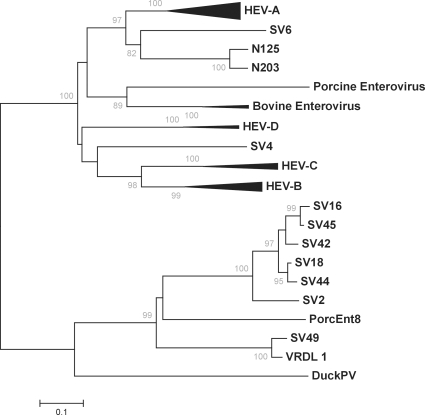
Phylogenetic analysis of likely simian Sapelovirus. Unrooted neighbor-joining phylogenetic relationships based on alignment of VP1 amino acid sequences, including partial simian virus genomes. Bootstrap analysis with 1000 pseudo-replicates was utilized.

Viral sequences obtained from sample VRDL1 represent a highly divergent picornavirus most closely related to simian picornavirus 1 (SV2), a member of the putative “sapelovirus” genus [22], with only 63% deduced amino acid identity within the originally sequenced VP2, VP3 and VP4 regions. Sub-genomic fragments were linked by PCR and both 5′- and 3′-RACE were utilized to acquire the complete VRDL1 polyprotein sequence which exhibited 80% amino acid identity to SV2. Except for the complete genome of SV2, sequence of simian sapeloviruses polyproteins currently are limited to VP1 and 3D regions [23],[24]. To characterize the relation of VRDL1 within the sapeloviruses, the deduced amino acid sequence for VP1 was used for phylogenetic reconstruction (**Fig. 2**). VRDL1 VP1 clustered tightly to SV49 VP1, a divergent member within the sapelovirus cluster; the two sequences showing 91% amino acid identity (**Fig. 2**). Similar results were seen for partial sequence data within 3D (data not shown) [24]. We therefore report the complete polyprotein sequence of SV49 strain VRDL1 (SV49-VRDL1) which is sufficiently divergent from SV2, with amino acid identities ranging from 52.3% in VP1 to 98.9% within the conserved 3D polymerase, to be considered the prototypic genome sequence for a second simian sapelovirus genotype.

### Reoviruses

#### Eyach virus isolated in United States

**Figure 3:**
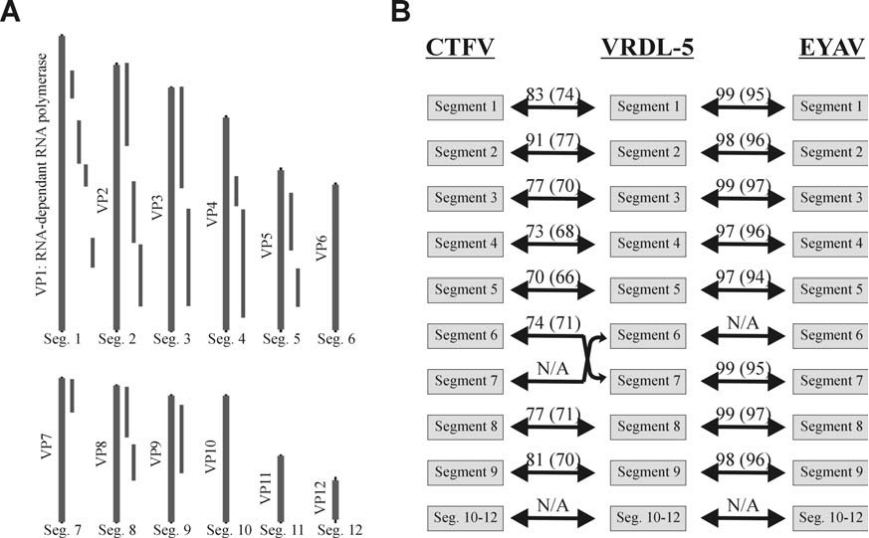
Eyach Virus. (A) Positions of VRDL4 fragments amplified relative to segmented *Coltivirus* genome. (B) Pairwise amino acid (nucleotide) percent identities between VRDL4 and CTFV and VRDL4 and EYAV for each segment.

The VRDL4 sample was originally derived from a pool of California mosquitoes collected in 1980 as part of the arbovirus surveillance program. Viral sequences from VRDL4 shared strong amino acid identity to members of the *Coltivirus* genus. Eyach virus (EYAV), Colorado tick fever virus (CTFV), and CTFV-related viruses are the sole members of the *Coltivirus* genus. Partial sequencing of segments 1, 2, 3, 4, 5, 7, 8, and 9 all shared greater than 94% nucleotide identity to the prototype strain of EYAV, originally identified in *Ixodes ricinus* ticks in Germany in 1976 [25] (**Fig. 3a**). To date, the reported geographical distribution of EYAV has been limited to Europe, while CTFV (and related strains) have been isolated in the United States [26]. In a phylogenetic tree based on concatenated VP1 amino acid sequence, VRDL4 paired with EYAV, with strong bootstrap support (data not shown). Overall amino acid and nucleotide identities across all segments sequenced suggest VRDL4 represents the first isolation of EYAV (EYAV strain VRDL4) from the United States. (**Fig. 3b**).

#### Novel Orthoreovirus

**Figure 4:**
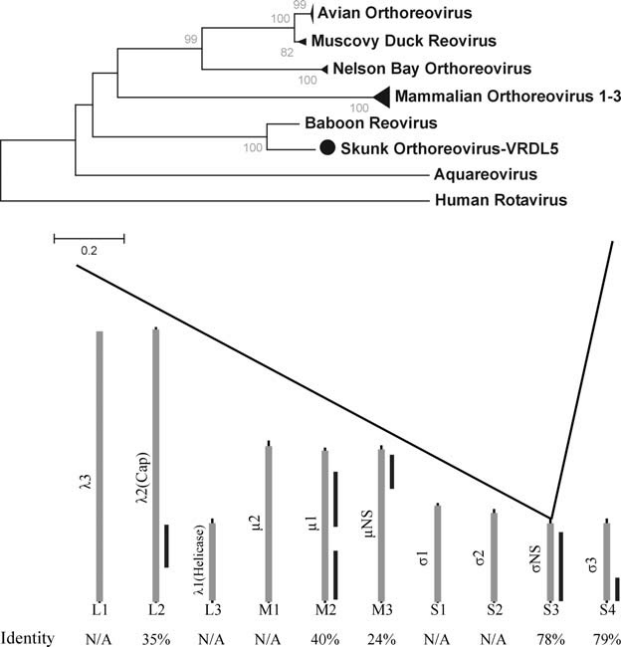
Novel Orthoreovirus. Alignment of six VRDL5 fragments amplified through viral discovery approach to ten genomic fragments of model orthoreovirus with amino acid identities (bottom). Neighbor-joining tree from orthoreovirus sigmaNS protein with bootstrap values from 1000 pseudo-replicates (top).

VRDL5, isolated from skunk brain tissue intracerebrally inoculated into suckling mice, displayed significant sequence identity to several fragments of mammalian orthoreoviruses. Sequence from VRDL5 was obtained for 5 of the 9 segments, with amino acid identities to known mammalian orthoreoviruses ranging from 23% to 79%. The greatest amount of coverage (979bp of ∼1150bp) was obtained for the S3 fragment encoding the sigma NS protein. This fragment shared approximately 78% amino acid identity with baboon reovirus (**Fig. 4**). For the S4 segment, VRDL sequences also paired with baboon reovirus. To date, no baboon reovirus class L or M segments have been published. VRDL5 fragment M2 shared closest sequence identity to avian orthoreovirus, while both L2 and M3 were weakly similar to mammalian orthoreoviruses. We propose VRDL5 represents a new orthoreovirus, tentatively named “skunk orthoreovirus”, which shares greatest similarity to baboon reovirus.

#### Novel Orbivirus

**Figure 5:**
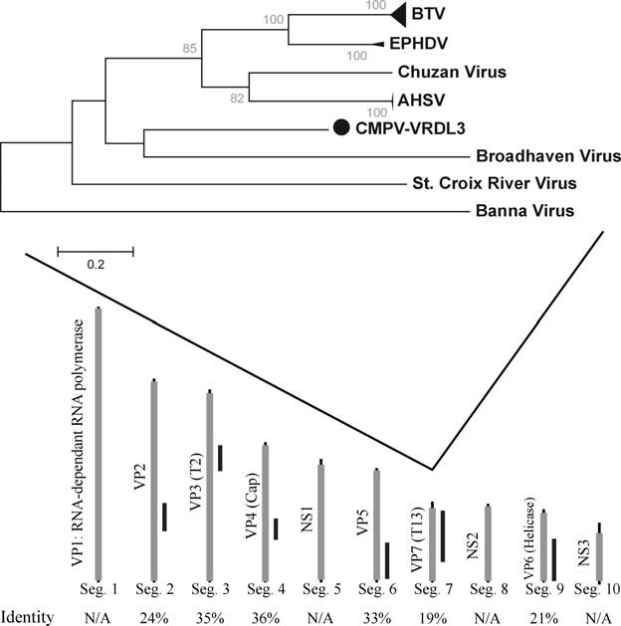
Novel Orbivirus. Alignment of VRDL3 fragments identified to genomic segments of model orbivirus sequence with amino acid identity percentages (bottom). Orbivirus VP7 amino acid neighbor-joining phylogenetic tree with bootstrap values from 1000 pseudo-replicates (top).

Sample VRDL3 consisted of brain tissue from suckling mice that had been inoculated intracerebrally with pooled *Culex tarsalis* homogenate. Viral sequences from VRDL3 displayed closest similarity to members of the Orbivirus genus in the *Reoviridae* family. Sequence from six of the ten total segments of Orbiviruses displayed weak amino acid identities (19–36%) to subcloned VRDL3 fragments (**Fig. 5**). Amino acid sequence from VP7, a structural protein, which along with VP3 forms the viral capsid [27], is the protein primarily used for phylogenetic analysis of Orbiviruses and exhibits the highest level of conservation within members of the Orbivirus genus with intraspecies amino acid identities between 83–99% [28],[29],[30]. VRDL3 VP7 exhibited only 19% amino acid identity to the closest Orbiviruses, and phylogenetic analysis revealed a deep, weakly bootstrap-supported pairing to the Great Island Broadhaven virus (**Fig. 5**). We propose VRDL3 is a novel virus and propose the name California Mosquito Pool Virus (CMPV), in accordance with traditional nomenclature of Orbiviruses.

Several members of the Orbivirus genus, including Bluetongue virus (BTV) and epizootic hemorrhagic disease virus (EPHDV), contain a conserved RGD motif located between positions 168 to 170 of VP7 shown to be necessary for attachment to *Culicoides* cells [31]. This motif is missing in Orbiviruses transmitted by mosquitoes, (e.g. Yunnan virus), or ticks [e.g. Broadhaven (BRDV) and St. Croix River virus (SCRV)]. CMPV VP7 contained a RHD motif, similar to the conserved RGD motif of BTV and EPHDV, despite being isolated from *Culex* mosquitoes. BTV-10 VP7 crystal structure [32] and functional analysis also have revealed several conserved residues within nine alpha helices involved in formation of the icosahedral viral core. Predicted secondary structure from CMPV VP7 partial sequence reveals the presence of the four C-terminal alpha helices (data not shown) and includes conserved residues corresponding to BTV-10 D318 and Y271, the latter shown to be necessary for stable trimerization and particle formation [27].

## Discussion

Genetic and antigenic diversity and the presence of un-characterized viruses can confound virus identification using species or serotype specific reagents. PCR tests generally exhibit increased sensitivity relative to antibody based antigen detection. However, viral genetic diversity can prevent the detection of viral variants. Use of degenerate consensus primers targeting conserved viral regions can improve detection of variants, but often at the cost of decreased sensitivity.

In this study, we circumvent the need for prior knowledge of viral sequence using sequence independent amplification of nuclease protected viral particles [15]. We tested samples of ground mouse muscle or brain that had been inoculated with suspected infectious material including human feces, ground mosquito pools, and sewer effluent. Processing of samples and identification of five previously undetected viruses was achieved in under a week. From each of the samples tested, a single viral species was identified. Typically, the majority of clones sequenced contained viral sequence. Of the five viruses amplified two were picornaviruses. One picornavirus was a borderline new serotype of HEV-C, most closely related to CoxA22, and the other was a member of the putative sapelovirus genus, SV49 strain VRDL1. Three reoviruses were identified: one was a very close match to Eyach virus, while the other two were sufficiently divergent from members of the O*rbivirus* and O*rthoreovirus* genera to be candidates for new species with the *Reoviridae* family. The Eyach virus, derived from pooled mosquitoes collected in 1980 is the first reported case of Eyach detection in the US. Eyach virus previously was thought to be restricted to Europe, while the related coltivirus species CTFV was found only in the US [26]. Current prevalence in US mosquitoes remains to be determined.

### Identification of novel viruses

We consider it unlikely that SV49-VRDL1, a simian sapelovirus, was derived from the sewer effluent used as inoculum, but instead was likely a contamination of lengthy *in vitro* manipulation in primary monkey kidney cells prior to mouse inoculation. Isolation of several simian enteroviruses in the early development and testing of poliovirus vaccine in primary monkey kidney cells illustrates the hazard of cell culture introducing viral contaminants [33],[34],[35],[36]. Regardless of its origin, we report the complete genome sequencing of SV49-VRDL1, a second simian sapelovirus exhibiting an overall amino acid identity of 80% and 77% nucleotide identity compared to SV2.

Orthoreoviruses currently consist of four species: mammalian orhtoreovirus (MRV), avian orthoreovirus (ARV), Nelson Bay orthoreovirus (NBV), and Baboon reovirus (BRV), although complete genetic sequence of BRV has not been described. Additionally, incomplete genomes from three tentative species have been described recently in reptiles [37]. Depending on the host and viral species, orthoreoviruses can cause respiratory illness, diarrhea, and severe neurological disorders. Infection in humans is generally benign, but can cause respiratory or gastrointestinal illness, with increased severity in infants [38],[39]. Demarcation of orthoreovirus species is based on sequence divergence, host species, and pathogenesis.

To determine if the skunk orthoreovirus identified represents a new species of orthoreovirus or a novel variant of the baboon reovirus species, intraspecies and interspecies sequence diversity within the orthoreovirus genus were examined. Deduced sigma non-structural protein sequences share 83–98% amino acid identity within avian orthoreoviruses (ARV) and 73%–94% identitiy within mammalian orthoreoviruses (MRV) [40],[41]. Pair-wise amino acid comparisons between AVR, BRV, and MRV species exhibit identities between 18–53% [37],[40]. The skunk orhtoreovirus S3 fragment shares 78% amino acid identity to baboon reovirus, falling into a gray zone with regard to species demarcation. Similarly, the BRV and skunk orthoreovirus S4 segment encoding the fusion-associated small transmembrane protein exhibit 79% amino acid identity. Due to the paucity of known baboon reovirus sequences in Genbank we cannot make direct comparisons with class L and M segments [40],[42]. Biologically, the tropism of BRV and skunk orthoreovirus differ at the species level, but both were isolated from brain tissue. In total, these data indicate the skunk orthoreovirus is likely to be a classified as either a new highly divergent serotype within the baboon reovirus species or a new species.

BTV, AHSV, and EPHDV within the *Orbivirus* genus are emerging pathogens of livestock with significant economic impact. BTV exhibits only rare cases of morbidity in cattle but is lethal in up to 70% of infected sheep and in all cases causes significant morbidity [43]. Less is known regarding the pathogenesis, prevalence, and complete sequence data for other species within the *Orbivirus* genus, including BRDV, SRCV, Yunnan and Palyam viruses. Differentiation of species is largely based upon vector transmission, overall amino acid identity and phylogenetic analysis of viral core (segment 7, VP7) or the RNA-dependent RNA polymerase (segment 2) [29],[44],[45]. Within the *Orbiviruses*, amino acid identities between serotypes, such as BTV-1 through BTV-4 are typically above 80% [28],[30],[46] or 94% in EPHDV [29] within segments 2 and 7. Divergence between BTV and the less well characterized Yunnan, SRCV or BRDV exhibits between 21–37% identity within segment 2 and 7 [44],[45],[47]. The six fragments of CMPV isolated all exhibit amino acid identities below 36% to all described Orbiviruses, qualifying it as a new Orbivirus species. Transmission of *Orbiviruses* involves three primary vectors; midges (BTV, AHSV), ticks (BRDV, SCRV) and mosquitoes (Yunnan virus). While phylogenetic analysis of VP7 indicated CMPV branches with very low bootstrap support with BRDV, CMPV was isolated from *C. tarsalis* mosquito pools while BRDV is tick-borne [48]. Taken together these data indicate CMPV may be the founding member of a new species within the *Orbivirus* genus.

These results indicate that known and novel viruses can be readily characterized using limited sequencing, provided that they are in the high concentration expected from affected tissues of recently inoculated mice showing acute pathologies. Application of such techniques to hard-to-type isolates may therefore accelerate the identification of new viral species.
